# Prediction of non-intuitive metabolic targets with bayesian metabolic control analysis to improve 3-hydroxypropionic acid production in *Aspergillus niger*

**DOI:** 10.3389/fbioe.2026.1754875

**Published:** 2026-02-17

**Authors:** Ziyu Dai, Jeremy D. Zucker, Yichao Han, Shant Mahserejian, Joseph Cottam, Nathalie Munoz, Yuqian Gao, Guoliang Yuan, Beth A. Hofstad, Jon K. Magnuson, Joonhoon Kim, Young-Mo Kim, Kristin E. Burnum-Johnson, Kyle R. Pomraning

**Affiliations:** 1 DOE Agile BioFoundry, Emeryville, CA, United States; 2 Energy and Environment Directorate, Pacific Northwest National Laboratory, Richland, WA, United States; 3 Earth and Biological Sciences Directorate, Pacific Northwest National Laboratory, Richland, WA, United States

**Keywords:** 3HP, 3-hydroxypropionic acid, Aspergillus niger, bayesian metabolic control analysis, non-intuitive, prediction

## Abstract

Development of efficient bioconversion processes is limited by the ability to predictably improve metabolic flux. Here we deployed Bayesian Metabolic Control Analysis as a platform to integrate multi-omics data with metabolic modeling and evaluated its ability to predict genetic interventions that improve metabolic flux. Global Metabolomics and proteomics data was collected from 17 *Aspergillus niger* strains engineered to produce the platform biochemical 3-hydroxypropionic acid from which seven actional genetic interventions were predicted from significant flux control coefficients. Of the suggested genetic interventions, two were present within the intuitively designed strains used for training (malonic semialdehyde dehydrogenase and pyruvate carboxylase) while five predicted targets were present within non-intuitive areas of the metabolic network including 5-formyltetrahydrofolate deformylase and four mitochondrial enzymes, alcohol dehydrogenase, succinyl-CoA ligase, aspartate aminotransferase, and malate dehydrogenase. Six of the targets were validated in the highest performing 3-HP strain used for multi-omics data generation which contained a prior disruption of the highest scoring target malonic semialdehyde dehydrogenase. Predicted directional perturbation of five of the six tested targets significantly improved titer and rate of 3-HP production and two significantly improved yield. The greatest improvements were observed following disruption of the non-intuitive target succinyl-CoA ligase which increased titer by 39% and yield by 29% (to 20.4 g/L 3-HP and 0.31 g 3-HP/g glucose) over the strains used for training. This study demonstrates the utility of Bayesian Metabolic Control Analysis and highlights the ability to predict meaningful genetic targets in unexpected areas of metabolism to improve engineered strains for bioconversion.

## 1 Introduction

Biological production of 3-hydroxypropionic acid (3-HP) from CO_2_ derived feedstocks including lignocellulosic material is a promising route to reduce emissions during commodity scale chemical production (Bhagwat et al., 2021; Karp et al., 2017). 3-HP is a 3-carbon building block that can be used as a chemical precursor for production of a variety of valuable polymers through catalytic dehydration to acrylic acid (Li et al., 2018), or acrylonitrile through nitrilation (Karp et al., 2017) or other commodity scale chemicals including acrylamide, 1,3-propanediol, and methyl acrylates (Werpy and Petersen, 2004). The total market size for commodity scale chemical products that can be derived from 3-HP is well in excess of a million metric tons per year and offers a substantial opportunity to reduce emissions by transitioning from petrochemical to biologically derived feedstocks for polymer markets (Wang et al., 2023). Reducing emissions during production of 3C polymers will require low-carbon-intensity 3-HP at or near cost-parity to petroleum derived propylene from which acrylic acid is derived by oxidation. To achieve this will require production of 3-HP from inexpensive feedstocks at near theoretical yield while minimizing fermentation and downstream costs by using an acidic bioconversion host (Bhagwat et al., 2021; Werpy and Petersen, 2004).

Commercial interest in production of 3-HP as a platform chemical is long-standing with a well-developed patent landscape around the efficient production of 3-HP from sugars. Significant efforts have been focused on development of 3-HP pathways in model hosts including the yeast *Saccharomyces cerevisiae* and the bacterium *Escherichia coli*, which have achieved yields of 0.31 and 0.30 g/g from glucose in neutral to mildly acidic conditions (Yu et al., 2022; Zhang et al., 2023). To reduce the cost of biomanufacturing via 3-HP as a platform biomolecule basic process requirements include (1) use of inexpensive readily existing feedstocks, (2) establishment of an acidic fermentation to minimize contamination, gypsum formation, and down-stream separation costs, (3) use of an industrial fungal host to enable a low pH fermentation, avoid phage induced process failure, and minimize feedstock nutrient additives, and (4) high titer, rate, and yield metrics. To accommodate all of the necessary process constraints we have focused on development of a theoretically high yield pathway in the acidophilic fungus *A. niger* which is capable of converting wide ranging lignocellulosic and waste feedstocks to 3-HP via the β-alanine bioconversion pathway, selected because it exhibits less dependency on oxygen uptake at high yields (Borodina et al., 2015) and operates in *Aspergillus niger* at acidic pH (Dai et al., 2023).

To increase the yield of 3-HP from sugars in *A. niger* fermentation we sought to adopt an approach that would generate predictions of reactions that control metabolic flux. To accomplish this, we leveraged Bayesian Metabolic Control Analysis (BMCA) to infer metabolic kinetics from genome-scale multi-omics data (McNaughton et al., 2021; St John et al., 2019). BMCA is a probabilistic framework for calculating metabolite and flux control coefficients from experimental data. Unlike traditional Metabolic Control Analysis (MCA), which provides point estimates, BMCA yields a posterior distribution for control coefficients, allowing for the quantification of uncertainty and the integration of diverse omics datasets (e.g., proteomics, metabolomics, fluxomics) to constrain the model. The BMCA framework employs the lin-log approximation of enzyme kinetics. This assumption linearizes the non-linear relationship between reaction rates (fluxes) and metabolite concentrations in logarithmic space, making the elasticities locally constant. This approach allows the system to be described by linear equations involving these coefficients, simplifying the calculation of steady-state to a single linear solve, while remaining accurate for small perturbations around a reference steady state. Here, we applied the BMCA framework with proteomics and metabolomics datasets to predict genes that control yield of 3-HP from sugars in engineered *A. niger* strains.

## 2 Materials and methods

### 2.1 Strain maintenance and cultivation

*Escherichia coli* strain Top10 was used for routine plasmid DNA preparation. *Aspergillus niger* strains are all derived from ATCC11414 from the American Type Culture Collection (Rockville, MD, United States) and were grown on complete medium (CM) or potato dextrose agar (PDA) plates (JW and LL, 1991) at 30 °C for culture maintenance and spore preparation. About 5 × 10^4^ to 5 × 10^5^ spores were inoculated on CM agar (Petri dish) plates and incubated for 4 days at 30 °C. Spores were harvested by washing with 5–10 mL sterile 0.4% Tween H_2_O and pelleted by centrifugation at 2,500 *g* for 5 min. The spores were re-suspended in the sterile 0.4% Tween H_2_O and enumerated with a hemocytometer. The spore suspensions were used for agar-plate or liquid cultures. The shake flask cultures were performed at 30 °C, 200 RPM in a New Brunswick Innova 44R stackable incubator shaker (Eppendorf, Endfield, CT, United States) with Pyrex 125 mL or 250 mL glass Erlenmeyer flasks which were prepared by filling with 5% Contrad 70 (Decon Labs, Inc., King of Prussia, PA, United States) and soaked overnight to remove any potential residues on the inside surface of flasks prior to general dishwashing. Silicon sponge closures were used for all flask cultures. For 3-HP production, sterile modified production medium B (mRDM; 100 g/L glucose, 5 g/L (NH_4_)_2_SO_4_, 0.11 g/L KH_2_PO_4_, 2.08 g/L MgSO_4_.7H_2_O, 0.13 g/L CaCl_2_.2H_2_O, 0.074 g/L NaCl, 4 mg/L CuSO_4_.5H_2_O, 110 mg/L FeSO_4_.7H_2_O, 14 mg/L MnCl_2_.4H_2_O, 26 mg/L ZnSO_4_.7H_2_O) (Dai et al., 2023) was used for cultivations.

### 2.2 Strain construction

Strains were constructed as described previously (Dai et al., 2023; Pomraning et al., 2021) with additional strains containing intermediatory or combinatorial expression of transgene vectors included here to provide diversity in expression of 3-HP pathway components. In this study, all transgene expression cassettes were prepared with Gibson assembly master mix (NEB, Ipswich, MA, United States) and the DNA fragments were isolated by PCR with Phusion high-fidelity DNA polymerase (Thermo Fisher Scientific, Waltham, MA, United States). Strain ABF_008348, the highest performing strain in the reference dataset was used as the host for validation of target genes. In this background, targets for overexpression (purU, mdhA, pyc2, and aat1) were constructed using the several strong constitutive promoters (*cox1*, *mbfA*, *tef1*, and *ubi4*) and integrated into the genome. Strains containing deletions of *adhD*, *suclg1*, and *suclg2* were constructed using CRISPR-Cas9 system for single gene editing in *A. niger* (Yuan et al., 2024) to create large indels in the genes targeted for deletion. Protoplast preparation and chemical-mediated transformation followed the method described previously (Dai et al., 2013). All strains used in this study are shown in Table 1. Plasmid and strain construction is described in further detail in Additional File 1.

**TABLE 1:** Strains used in this study.

Strain	Genotype	Purpose	References
ABF_008340	wild-type	Omics Data	Perlman et al. (1946)
ABF_008343	[βAI-3HP]+	Omics Data	Dai et al. (2023)
ABF_008344	[βAI-3HP]+, aat1+	Omics Data	Dai et al. (2023)
ABF_008345	[βAI-3HP]+, pyc2+	Omics Data	Dai et al. (2023)
ABF_008346	[βAI-3HP]++, pyc2+	Omics Data	this work
ABF_008347	[βAI-3HP]+, pyc2+, ΔoahA	Omics Data	Dai et al. (2023)
ABF_008348	[βAI-3HP]+, pyc2+, Δald6a	Omics Data	Dai et al. (2023)
ABF_008349	[βAI-3HP]+, pyc2+, Δald6b	Omics Data	Dai et al. (2023)
ABF_008351	[βAI-3HP]+, pyc2+, Δuga2	Omics Data	Dai et al. (2023)
ABF_008354	[βAI-3HP]+, pyc2+, aat2+	Omics Data	this work
ABF_008355	[βAI-3HP]++, pyc2+, aat2+	Omics Data	this work
ABF_008356	[βAI-3HP]+, pyc2+, Δald6a, aat2+	Omics Data	this work
ABF_008897	[βAI-3HP]++, pyc2+, mct1+	Omics Data	this work
ABF_008898	[βAI-3HP]+, pyc2+, mct1+	Omics Data	Dai et al. (2023)
ABF_008899	[βAI-3HP]++, pyc2+, ΔoahA	Omics Data	this work
ABF_015658	[βAI-3HP]++, pyc2+, uga2+	Omics Data	this work
ABF_009101	[βAI-3HP]+, pyc2+, aat2+	Omics Data	this work
ABF_011231	[βAI-3HP]+, pyc2+, Δald6a, aat2+	Validation	this work
ABF_011232	[βAI-3HP]+, pyc2+, Δald6a, aat1+	Validation	this work
ABF_011234	[βAI-3HP]+, pyc2+, Δald6a, mdhA+	Validation	this work
ABF_011236	[βAI-3HP]+, pyc2+, Δald6a, purU+	Validation	this work
ABF_011233	[βAI-3HP]+, pyc2+, Δald6a, pyc2+	Validation	this work
ABF_011239	[βAI-3HP]+, pyc2+, Δald6a, ΔadhD	Validation	this work
ABF_011240	[βAI-3HP]+, pyc2+, Δald6a, ΔiscA	Validation	this work
ABF_011241	[βAI-3HP]+, pyc2+, Δald6a, ΔiscB	Validation	this work

### 2.3 Metabolome and proteome analysis

For quantification of extracellular metabolites in the spent medium samples were analyzed using high performance liquid chromatography (HPLC) equipped with a Waters 2,414 refractive index detector. A Bio-Rad Aminex HPX-87H ion exclusion column (300 mm × 7.8 mm), heated to 65C was used for analyte separation. Sulfuric acid (0.0045 M) was used as eluent at a flow rate of 0.55 mL/min. Intracellular metabolite extracts were completely dried under vacuum to remove moisture and chemically derivatized. Briefly, the extracted metabolites were derivatized by methoxyamination and trimethylsilyation (TMS), then the samples were analyzed by GC-MS. Sample preparation, instrument acquisition and data analysis was performed as previously reported (Pomraning et al., 2021; Kim et al., 2015). Global and targeted proteomics was performed as in previously established LC-MS/MS methods and data analysis workflows (Pomraning et al., 2021), except slight adjustments to the mass spectrometry acquisition settings in global proteomics. In global proteomics, peptide digests were analyzed using a Q Exactive Plus mass spectrometer (Thermo Fisher Scientific) in data-dependent acquisition mode. Mass spectrometer settings were as following: full MS (AGC, 3 × 10^6^; resolution, 70,000; m/z range, 300–1800; maximum ion time, 20 m); MS/MS (AGC, 1 × 10^5^; resolution, 17,500; m/z range, 200–2000; maximum ion time, 50 m; TopN, 12; isolation width, 1.5 Da; dynamic exclusion, 30.0 s; collision energy, NCE 30).

### 2.4 Bayesian inference for kinetic parameter estimation

BMCA was performed following established protocols (McNaughton et al., 2021; St John et al., 2019). A reduced metabolic model of *A. niger* was constructed by integrating the central metabolism of iJB1325 (Brandl et al., 2018) and beta-alanine pathway for 3-HP production and removing reactions with zero flux at the reference state. The resulting model comprised 172 reactions and 171 metabolites. Proteomics and metabolomics measurements from 51 samples representing 17 distinct strains were used as the observed data. Strain-specific uptake and excretion rates were calculated for measured extracellular metabolites. These rates were further used to calculate internal fluxes with global proteomics as constraints with a proton export objective using E-Flux2 (Kim et al., 2016). At steady state, the metabolic reaction rate ( v ) can be expressed as a function of enzyme concentrations ( e ), internal and external metabolite concentrations ( x and y ) as follows:

$$v=\text{diag}\left(v^{*}\frac{e}{e^{*}}\right)\left(1_{n}+\varepsilon_{x}^{*}\log\frac{x}{x^{*}}+\varepsilon_{y}^{*}\log\frac{y}{y^{*}}\right)$$

where the asterisks ( * ) denote quantities at a defined reference state, $\varepsilon_{x}^{*}$ and $\varepsilon_{y}^{*}$ are kinetic parameters called metabolite elasticity matrixes, which respectively quantify the sensitivity of reaction rates to changes in internal and external metabolite concentrations. This lin-log approximation is valid near the reference state and enables efficient parameter estimation (St John et al., 2019).

Posterior distributions of the model parameters were inferred using automatic differentiation variational inference (ADVI), as implemented in the PyMC3 Python library. The model was optimized using the Adagrad optimizer until convergence of the negative evidence lower bound score. Using the resulting parameterized kinetic model, we computed flux control coefficients (FCCs), which quantify the sensitivity of steady-state fluxes to perturbations in enzyme levels at the reference state. FCCs were considered significantly different from zero if their 95% highest posterior density intervals from the posterior distribution do not overlap with zero. Github repositories for the *Aspergillus*-specific analysis are available at https://github.com/agilebiofoundry/aspergillusq4milestone and for the BMCA at https://github.com/agilebiofoundry/bayesian-metabolic-control-analysis.

## 3 Results

### 3.1 Multi-omic analysis of *Aspergillus niger* strains engineered to produce 3-HP

**FIGURE 1:**
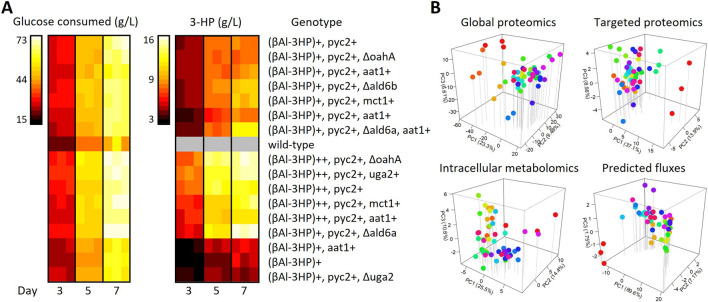
Metabolic analysis of 3-HP production in *Aspergillus niger*. **(A)** Time-series production of 3-HP from glucose in diverse strains. **(B)** Samples for multi-omics analysis were collected in triplicate on day 5 and metabolic fluxes predicted with consumption, production, and growth as constraints. Abbreviations: βAla-3HP, beta-alanine heterologous pathway consisting of *Tc*PAND, *Bc*BAPAT, and *Ec*HPDH; AAT1, aspartate aminotransferase; ALD6, malonic semialdehyde dehydrogenase; *Bc*BAPAT, beta-alanine pyruvate aminotransferase; *Ec*HPDH, 3-hydroxypropanoate dehydrogenase; MCT1, monocarboxylate transporter; OAHA, oxaloacetate hydrolase; PYC2, pyruvate carboxylase; UGA2, succinate semialdehyde dehydrogenase; *Tc*PAND, aspartate decarboxylase.

Metabolomic and Proteomic analysis was conducted to identify reactions controlling flux in *A*. *niger* strains engineered to produce 3-HP. Strains were constructed or selected from a previous studies (Dai et al., 2023) to perturb flux through the beta-alanine 3-HP biosynthetic pathway enabled by overexpression of three heterologous enzymes, *Tc*PAND, *Bc*BAPAT, and *Ec*HPDH (Borodina et al., 2015). Previously identified genetic targets including *oahA*, *uga2*, *ald6a*, *ald6b*, *pyc1*, *aat1*, *mct1*, and the heterologous beta-alanine pathway genes (Dai et al., 2023; Pomraning et al., 2021) were disrupted and overexpressed to establish a dataset spanning a wide range of 3-HP productivity levels amenable to learn from (Table 1). All strains were cultivated in mRDM for 7 days with extracellular metabolites and biomass collected at days 3, 5, and 7 to provide boundary constraints for flux balance analysis (Figure 1). Cell pellets collected on day 5 were extracted using MPLEx (Nakayasu et al., 2016) for mass-spec based multi-omics assays. Data from 17 strains in triplicate was collected for 55 intracellular metabolites (Kim and Heyman, 2018) and 3,814 proteins using global untargeted methods and 59 proteins selected for quantitatively precise measurement by selected reaction monitoring (SRM) based targeted proteomics (Gao et al., 2020) (Additional file 2).

Intracellular reactions fluxes were predicted using a reduced metabolic model (172 reactions, 171 metabolites) adapted from a genome-scale metabolic model of *A. niger* (Brandl et al., 2018) that contained only non-zero fluxes at the reference state. The spent media, time, and biomass at collection were used to construct a simple exponential growth model of the organism and estimate strain-specific uptake and excretion rates for key measured extracellular metabolites. These rates were further used to calculate internal fluxes with global proteomics as constraints using E-Flux2 (Kim et al., 2016).

### 3.2 Bayesian metabolic control analysis to predict reactions controlling metabolic flux

We next employed the BMCA methodology developed to predict how cellular kinetics respond to genetic changes (McNaughton et al., 2021; St John et al., 2019). In BMCA, a low-fidelity kinetic model of microbial metabolism is constructed leveraging linear-logarithmic kinetics. With known kinetic parameters, a kinetic model enables the expected steady-state internal metabolite concentrations and metabolic fluxes to be estimated as a function of enzyme expression and media conditions. With measurements of both the input variables (extracellular metabolite concentrations and enzyme expression) and the output variables (steady-state fluxes and internal metabolite concentrations), posterior distributions in the kinetic parameters that are consistent with the observed data can then be estimated.

**FIGURE 2:**
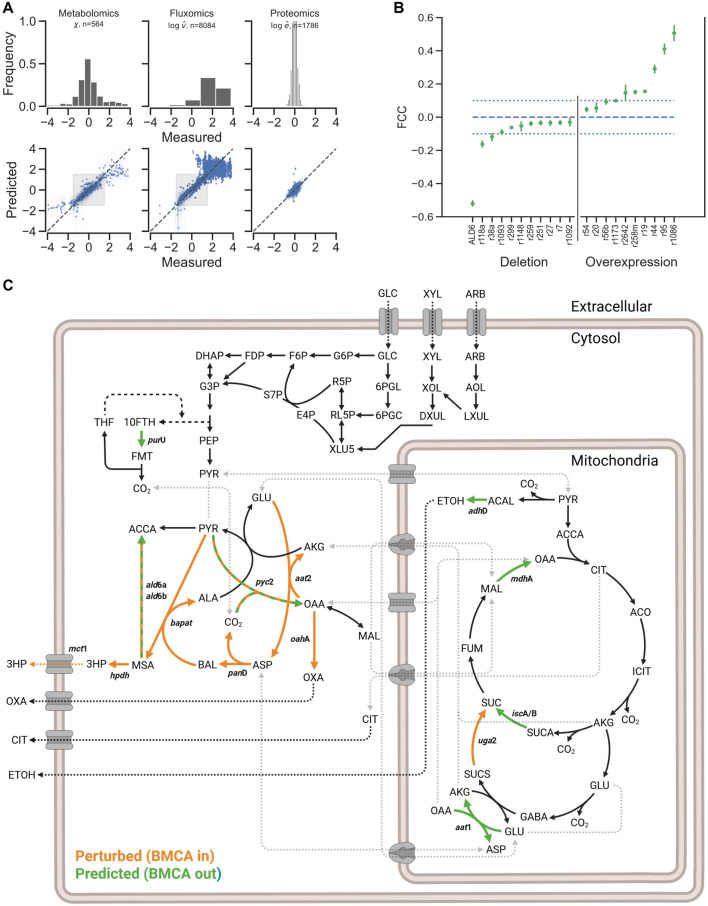
Bayesian metabolic control analysis. **(A)** Posterior predictive distribution of the fitted model. The metabolomics (left), intracellular and extracellular fluxes (center) and proteomics (right) closely match the experimentally measured values. Fluxes are in units of mmol/gDCW*hr, while metabolomics and proteomics data are in log-transformed, dimensionless units relative to the reference strain. Shaded boxes indicate the unclipped region where measured data is near the reference state. **(B)** Posterior distributions in 3-HP flux control coefficients (FCC). The dotted lines are provided as a qualitative aid for selecting candidate targets. FCC’s whose credible interval crosses the dashed line are not considered a target. **(C)** Gene included in multi-omics analysis dataset (perturbed) and with significant FCCs (predicted). Abbreviations: 10-FTH, 10-formyltetrahydrofolate; 3HP, 3-hydroxyproptionic acid; ACAL, acetaldehyde; AKG, alpha-ketoglutarate; ACCA, acetyl-CoA; ALA, alanine; ASP, aspartate; BAL, beta-alanine; ETOH, ethanol; FMT, formate; GABA, gamma-aminobutyrate; GLC, glucose; GLU, Glutamate; MAL, malate; MSA, malonic semialdehyde; OAA, oxaloacetate; OXA, oxalate; PYR, pyruvate; SUC, succinate; SUCA, succinyl-CoA; SUCS, succinate semialdehyde.

Due to the size of the kinetic model considered, posterior distributions in kinetic parameters as a function of the observed data was estimated using automatic differentiation variational inference as implemented in the PyMC3 Python library. The model was optimized until convergence of the evidence lower bound score using the Adagrad optimizer. The posterior predictive distribution (PPD) of the model shows the ability of the model to reproduce the measured experimental data across different strains. The PPD of the fitted model closely reproduces the measured targeted proteomics, metabolite concentrations, and global proteomics-based Eflux2-predicted intracellular fluxes within the unclipped shaded region (Figure 2A). Outside this region, predicted metabolomics, proteins and fluxes were based on clipped measurements, hence the horizontal cluster of sample points.

With a kinetic model and estimated probability distributions in kinetic parameters, we then conducted the Metabolic Control Analysis portion of the BMCA framework. Here, the uncertainty in the estimated kinetic parameters is propagated to the metabolic design strategies suggested by Metabolic Control Analysis. In Figure 2B, we show a subset of the highest posterior density regions of flux control coefficients (FCCs) on 3-HP export calculated from the posterior distribution. A positive FCC indicates that an increase in the corresponding enzyme concentration will increase 3-HP flux, while a negative FCC indicates that a decrease in enzyme concentration will increase 3-HP flux, thus FCCs capture the systems-level regulation of changing enzyme concentration on steady-state metabolic flux.

### 3.3 Validation of reaction targets with metabolic engineering to increase 3-HP production

To evaluate use of the BMCA framework as a predictive modeling tool for synthetic biology applications we selected reactions to modify based on their associated FCC in the training data. We predicted that reactions with negative FCCs will increase flux through the 3-HP pathway when downregulated or deleted and reactions with positive FCCs will increase flux through the 3-HP pathway when enzymes associated with the reaction are over-expressed (Figure 2). The largest positive flux control coefficients include plasmas membrane nitrate transport (r1086, ProteinID 1189116), 5-formyltetrahydrofolate deformylase (r95, ProteinID 1182700), malate dehydrogenase (r44, ProteinID 1144118), pyruvate carboxylase (r19, ProteinID 1031996), and mitochondrial aspartate aminotransferase (r258 m, ProteinID 1184650). The most negative FCC’s include the putative reaction catalyzed by ALD6 (malonate semialdehyde + CoA + NAD(P)^+^ → Acetyl-CoA + CO_2_ + NAD(P)H, ProteinID 1182225) (Dai et al., 2023; Pomraning et al., 2021), mitochondrial alcohol dehydrogenase (r118a, ProteinID 1145368) and succinyl-CoA ligase (r38a, ProteinIDs 1145655 and 1141712).

**FIGURE 3:**
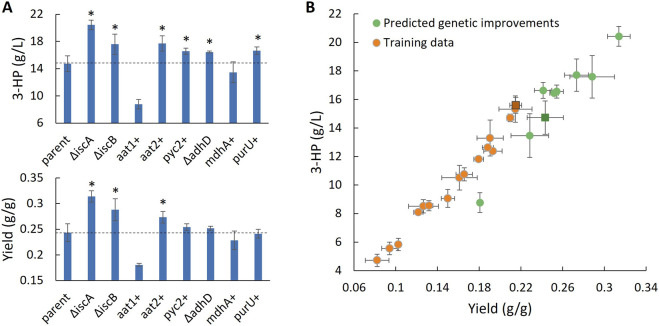
Validation of flux control predictions by genetic intervention. **(A)** 3-HP titer and yield metrics from glucose after 7 days of cultivation in selected transformants of high performing parent strain ABF_008348 (n = 3). Asterisks indicate significant improvements (p < 0.05). **(B)** 3-HP titer and yield in the training data and in strains produced to test for improvements in 3-HP production. The parent strain ABF_008348 used for testing is indicated as darkened squares in each dataset. Abbreviations: 3-HP, 3-hydroxyproptionic acid; aat1/2, aspartate aminotransferase; adhD, alcohol dehydrogenase; ald6, malonic semialdehyde dehydrogenase; iscA/B, succinyl-CoA ligase; mdhA, malate dehydrogenase; purU, 5-formyltetrahydrofolate deformylase; pyc2, pyruvate carboxylase.

We sought to delete or overexpress each predicted gene target in the highest performing 3-HP strain evaluated in the training set (strain ABF_008348). This strain has a preexisting *ald*6a disruption, the most strongly suggested deletion target, which we previously defined as a critical modification in 3-HP producing *Aspergilli* as well as *Rhodosporidium toruloides* (Dai et al., 2023; Pomraning et al., 2021; Liu et al., 2023). To evaluate alcohol dehydrogenase we selected a high-quality mitochondrial target, *adh*D (Protein ID 1145368) and assessed both subunits of the succinyl-CoA ligase enzyme. All the single gene overexpression targets were selected for evaluation except plasma membrane transport of nitrate as we had previously established that increasing the concentration of extracellular nitrogen promotes significantly higher yield and titer of 3-HP (Dai et al., 2023). Overexpression of mitochondrial aspartate aminotransferase AAT1 unexpectedly dramatically reduced 3-HP productivity, however, overexpression of a cytosolic variant (AAT2; ProteinID 1176455) improved both titer and yield of 3-HP. In total, six reactions with significant FCCs were evaluated for their impact on flux toward 3-HP. Predicted directional perturbation of five of the six reactions significantly improved titer and rate of 3-HP production and two significantly improved yield (Figure 3). Notably, disruption of the nonintuitive target succinyl-CoA ligase resulted in a 39% increase in titer (14.7–20.4 g/L 3-HP) and 29% increase in yield (0.24–0.31 g 3-HP/g glucose), highlighting the ability to predict meaningful genetic targets in unexpected areas of metabolism by integrating systems-level proteome and metabolome data with metabolic modeling and flux-omics.

## 4 Discussion

In conclusion, we developed the computational infrastructure to deploy BMCA to enable integration of systems-level multi-omics datasets with prior knowledge in the form of a genome-scale metabolic model, and metabolic flux predictions constrained by growth, consumption, and production rates. This approach enables the generation of small numbers of high-quality predictions to improve metabolic flux in organisms engineered to produce biochemicals at high yield. BMCA also requires less data collection than machine learning approaches due to the integration of a mechanistic model (Shin et al., 2026). While machine learning and artificial intelligence may expediate development of model microbes amenable to high-throughput genetic manipulation and rapid data-sparse phenotyping, data-rich approaches that globally characterize engineered microbes are needed for industrial hosts that are limited by genetic engineering or phenotyping rates, particularly when assessing bioprocesses at industrially relevant scales where even modest throughput is impractical.

## Data Availability

The original contributions presented in the study are included in the article/Supplementary Material, further inquiries can be directed to the corresponding author.
