# Supplementary material for: Prediction of non-intuitive metabolic targets with bayesian metabolic control analysis to improve 3-hydroxypropionic acid production in Aspergillus niger
Source: Front Bioeng Biotechnol. 2026 Feb 17;14:1754875. doi: 10.3389/fbioe.2026.1754875 (PMC12953433; doi:10.3389/fbioe.2026.1754875)
Supplement: Supplementary file 1 [file DataSheet1.pdf]

Additional File 1. Supplementary methods

Strain and plasmid construction

The plasmid 3HP4109 for uga2 disruption was prepared previously. The plasmid 3HP4136 containing the neomycin phosphotransferase II (*nptII*) marker gene, transgene expression plasmid 3HP4126 for *mct1* (*A. niger* monocarboxylate transporter 1) gene, and 3HP4145 of the new b-alanine pathway were prepared previously (Dai et al., 2023). The plasmid **pZD4123** for oahA gene disruption was prepared with the DNA fragments of 5'-oahA, nat1, and 3'-oahA isolated from plasmid DNA 4114 or *A. niger* genomic DNA with oligo pairs of 2426oahF1/2573oahR1, 2574nat1F/2575nat1R, and 2576oahF2/2431oahR2 and assembled into pBlueScript SK (-) linearized by restriction enzymes *Hin*dIII and *Pst*I. The transgene expression plasmid **pZD4125** for cyt*aat2* (cytosolic aspartate aminotransferase) gene was prepared by Gibson assembly. The fragments for *nat1* marker gene of the plasmid 3HP4114 reported previously (Dai), the *tef1* promoter, cDNA of cyt*aat2* gene, and *pgk1* transcriptional terminator of *A. niger* were isolated by PCR with the oligo pairs of 2583nat1F/2584nat1R, 2585tef1F/2247tef1R, 2248aat2F/2249aat2R, and 2550pgktF/2551pgk1tR and assembled into 3HP4136 linearized with restriction enzyme *Xho*I. The plasmid **pZD4134** for *uga2* (Mitochondrial succinate-semialdehyde dehydrogenase) transgene overexpressionwas constructed with the DNA fragments of *ubi4* promoter, *uga2* coding region and transcriptional terminator, and *mdh1* promoter isolated from *A. niger* genomic DNA and nptII coding region and nopaline synthase gene transcriptional terminator from pBI101 (Jefferson 1987, Plant Nolecular Biology Reporter 5:387-405) with the oligo pairs of 2658ubi4pF/2659ubi4pR, 2660uga2F/2661uga2R, 2662mdhpF/2663mdhpR, and 2664nptIIF/2665nptIIR, which were assembled into pBlueScript SK(-) linearized by restriction enzyme *Hin*dIII and *Pst*I. The plasmid **pZD4146** for mit*aat1* (mitochondrial aspartate aminotransferase) gene expression was prepared with the DNA fragments of *nat1* marker gene of 3HP4114, *ubi4* promoter and mit*aat1* of *A. niger* that were isolated with oligo pairs of 2583nat1F/2747nat1R, 2748ubi4pF/2749ubi4pR, and 2750maat1F/2751maat1R and assembled into 3HP4136 linearized with restriction enzyme *Xho*I. The transgene expression plasmid **pZD4154** for *pyc1* (pyruvate carboxylase) gene of *A. niger* was prepared with DNA fragments of *tef1* promoter and *pyc1* cDNA and its transcriptional terminator of *A. niger* using oligo pairs of 2795tef1pF/2796tef1pR, 2797pyc1F1/2798pyc1R1, and 2799pyc1F2/2800pyc1R2, which were assembled into the plasmid 3HP4136 linearized by restriction enzyme *Xho*I. To construct the plasmid **pZD4147**, the DNA fragments of *mbfA* promoter, *mdh1* coding sequence, and *pyc1* transcriptional terminator of A. niger were isolated by PCR with oligo pairs of 2913mbfApF/2919mbfApR, 2920mdhF/2921mdhR, and 2922pyct/2918pyctR and assembled into 3HP4136 linearized with restriction enzyme *Xho*I. The transgene expression plasmid **pZD4203** for *A. niger puru* (5-formyltetrahydrofolate deformylase) gene was prepared with the DNA fragments of *coxA* promoter and *puru* gene of A. niger and *Aspergillus pseudoterreus gpdA* transcriptional terminator, which were isolated by PCR with oligo pairs of 3041coxApF/3042coxApR, 3043puruF/4044puruR, and 3045apGpdAtF/2940apGpdAR and assembled into 3HP4136 linearized by restriction enzyme *Xho*I. The CRISPR-cas9 with double knockout construct pGY25, pGY26, and pGY27 for *adhD* (alcohol dehydrogenase D), *aclA* and *aclB* (ATP-citrate lyase/succinyl-CoA ligase, alpha- and beta subunits) were prepared by inserting the synthetic gBlocks Gene Fragments into the pGY18 (Yuan et al.) linearized by restriction enzyme *bsa*I with Golden Gate assembly.

The strain ABF_**008346** (3HP) was prepared by integrating the plasmid HP4145 linearized by XbaI into the chromosomes of the transgenic strain ABF_008345. The transgenic strain ABF_**008354** (aat2) was prepared by inserting the plasmid DNA pZD4125 linearized by the restriction enzymes *Xba*I *and Xho*I into the chromosomes of ABF_008345 strain. The same linearized plasmid DNA pZD4125 was integrated into the chromosomes of ABF_008345, ABF_008346, and ABF_008348 and generated the new transgenic strains of ABF_**008354**, ABF_**008355**, and ABF_**008356 (11231)**. The plasmid DNA pZD4146 (mitAAT1) linearized with restriction enzymes XbaI and XhoI was inserted into the chromosomes of *A. niger* strain ABF_008348 to form new ABF_**011232** strain. The strain ABF_**015658** was created by integrating linearized plasmid DNA pZD4134 with restriction enzymes *Xba*I and *Xho*I. The transgenic *A. niger* strains ABF_**008897** And ABF_**008898** were generated by inserting the linearized plasmid DNA 4126 (Dai et. al.) with restriction enzymes *Pvu*II and *Xba*I into the chromosomes of ABF_008345 and ABF_008346, respectively. The strain ABF_**008899** was created by oahA homologous disruption with plasmid DNA pZD4123 linearized by restriction enzyme PvuII. The transgenic strains ABF_**011233**, ABF_**011234**, and ABF_**011236** were generated by random insertions of the linearized plasmid DNA pZD4154, pZD4147, and pZD4203, respectively. Finally, the transgenic strains of ABF_011239, ABF_011240, and ABF_011241 were prepared by CRISPR-Cas9 double-knockout with the plasmid DNA pGY25, pGY26, and pGY27 in the ABF_008348 strain background.

Supplementary Table 1. Oligonucleotides used for plasmid and Strain Construction

Oligo name	Oligo nucleotides for **pZD4123**
2426oahF1	gaggtcgacggtatcgata**agctt**CTCAGCTGGGTGAAGAACAAC
2573oahR1	catcttctgtGTGATAGTGTTGGTCATGCTG
2574nat1F	cactatcacACAGAAGATGATATTGAAGGAGC
2575nat1R	gttagtatgCAGTAAGTAGAAAGCTTTGGG
2576oahF2	ctacttactgCATACTAACGGAAGGGTCAG
2431oahR2	gtggatcccccgggctgcaGTACCACGCAAGCTTCGATATG
	Oligo nucleotides for **pZD4125**
2583nat1F	aggtcgacggtatcgataACAGAAGATGATATTGAAGGAGC
2584nat1R	aggtcaacgCAGTAAGTAGAAAGCTTTGGG
2585tef1F	tacttactgCGTTGACCTCACAGGGATTTC
2247tef1R	aaggggcgaCTTACTGTTGTAGAAGATATCCGTTAG
2248aat2F	aacagtaagTCGCCCCTTTCCTCCTCTTC
2249aat2R	acagggcagTTATGAAGTCTCCCGAACTACG
2550pgktF	acttcataaCTGCCCTGTCGAGTAAGTAAATTTG
2551pgk1tR	tggatcccccgggctgcaACTACAGAGAGGAGCTGAAG
	Oligo nucleotides for **pZD4134**
2658ubi4pF	cgaggtcgacggtatcgataGGAGTTTCAACGGGTTCTATTTC

2659ubi4pR	atctctccatGTTGTAGAAGCGCAGTTAATG
2660uga2F	cttctacaacATGGAGAGATTTATTCGTCCAG
2661uga2R	ggagtcgttcCTGAGGATGAGAGCCAGTATTTG
2662mdhpF	tcatcctcagGAACGACTCCAGAAGTGACTAAG
2663mdhpR	gttcaatcatGGTGAAATTTGGGATTGTGAC
2664nptIIF	aaatttcaccATGATTGAACAAGATGGATTGC
2665nptIIR	agtggatcccccgggctgcaGACTCTGCTAAGCTATTCTTC
	Oligo nucleotides for **pZD4146**
2583nat1F	cgaggtcgacggtatcgataACAGAAGATGATATTGAAGGAGC
2747nat1R	tactccgaagCAGTAAGTAGAAAGCTTTGGG
2748ubi4pF	ctacttactgCTTCGGAGTAGCAACGAGTATTTTC
2749ubi4pR	tggacagcatGTTGTAGAAGCGCAGTTAATG
2750maat1F	cttctacaacATGCTGTCCACCCTCAGAGTC
2751maat1R	agtggatcccccgggctgcaCATACCTCTCTTTCTTCACTCGTG
	Oligo nucleotides for **pZD4154**
2795tef1pF	attgggtaccgggccccccctctagaGAGCATCATCCCATGATAG
2796tef1pR	ggggagcagcCTTACTGTTGTAGAAGATATCC
2797pyc1F1	caacagtaagGCTGCTCCCCGCCAGCCC
2798pyc1R1	tgcttacagaAGTGGTAGATGGCGTTGTCAGGGAGG
2799pyc1F2	atctaccactTCTGTAAGCAGGCCAAGAAG
2800pyc1R2	tattatcgataccgtcgaccAGATCTGGTACAGGCTAG
	Oligo nucleotides for **pZD4147**
2913mbfApF	attgggtaccgggcccccccCTGCAAGGGAGACTTGACGTTTC
2919mbfApR	gagcagcgaaCATTTTGAAGATGGATGAGAAGTCG
2920mdhF	cttcaaaatgTTCGCTGCTCGTCAGTCCCTC
2921mdhR	agccggtcagACCATTTAAGGGTTCTGCTTGACG
2922pyctF2	cttaaatggtCTGACCGGCTGCTAACATCTATTAG
2918pyctR	tattatcgataccgtcgaccCGCGTCTGGAAGGCTTTACG
	Oligo nucleotides for **pZD4203**
3041CoxApF	attgggtaccgggccccccc**tctaga**TGCAGGGAAGGTTGGAAATGG
3042CoxApR	tgtagctcatTGTCCTGGTGGGTGGGTTGC
3043purUF	caccaggacaATGAGCTACATCCTCACCCTC
3044purUR	ctgattcctaCCTAGTTGAAGACAACAGTCTTG
3045TpycF1	ttcaactaggTAGGAATCAGGACGGAATGTG
2940TgpdAR	tattatcgataccgtcgaccTGGATGCTGGAGCTCTTAAAC
	Oligo nucleotides for **pGY25, pGY26, & pGY27**
pGY25_sgRNA-1	AGCAACGCGTGAGGACACAG
pGY25_sgRNA-2	CAACTTCGAACTCATTGACG
pGY26_sgRNA-1	TCGCATTAATGGAGACACCA
pGY26_sgRNA-2	CTTGCTCAGCGTGGAAACTG
pGY27_sgRNA-1	CGTACCGAGGGACTTGGCAA
pGY27_sgRNA-2	ACGACCACCAGCAAGAACCT
